# Breakthrough Pain in Patients with Lung Cancer. A Secondary Analysis of IOPS MS Study

**DOI:** 10.3390/jcm9051337

**Published:** 2020-05-04

**Authors:** Sebastiano Mercadante, Francesco Masedu, Marco Valenti, Federica Aielli

**Affiliations:** 1 Main Regional Center for Pain, Pain Relief and Supportive/Palliative Care, La Maddalena Cancer Center, 90146 Palermo, Italy; 2 Department of Biotechnological and Applied Clinical Sciences, Section of Clinical Epidemiology and Environmental Medicine, University of L’Aquila, 67100 L’Aquila, Italy; francesco.masedu@univaq.it (F.M.); marco.valenti@univaq.it (M.V.); 3 Department of Medical Oncology, AUSL Teramo, 64100 Teramo, Italy; federicaaielli76@gmail.com

**Keywords:** lung cancer, cancer pain, breakthrough cancer pain, opioids

## Abstract

Aim: To characterize breakthrough cancer pain (BTcP) in patients with lung cancer. Methods: This was a secondary analysis of multicenter study of patients with BTcP. Background pain intensity and opioid dose were recorded. The number of BTcP episodes, their intensity, predictability, onset, duration and interference with daily activities were collected. Opioids used for BTcP, the mean time to meaningful pain relief after taking medication, satisfaction and adverse effects were assessed. Results: 1087 patients with lung cancer were examined. In comparison with other tumors, patients with lung cancer showed: higher background pain intensity (*p* = 0.006), lower opioid doses (*p* = 0.005), higher intensity of BTcP (*p* = 0.005), movement (79.5%) and cough (8.2%), as principal triggers for predictable BTcP (*p* < 0.009), larger BTcP interference with daily activity (*p* = 0.0001), higher use of adjuvants (*p* = 0.0001). No relevant differences in the other parameters examined were found. Conclusion: Patients with lung cancer have their own peculiarities, including higher basal and BTcP pain intensity and the use of more adjuvant drugs for background pain. The most frequent triggers for predictable BTcP are movement and cough. Future studies should be performed to analyze the prevalence of BTcP in patients with different lung cancers as well as the optimal management strategy for background pain and BTcP.

## 1. Introduction

Lung cancer is the most important cause of cancer-related death in the world. In 2016, about 220,000 new diagnoses of lung cancer were reported and about 150,000 of patients died for the disease. For every 100,000 people, 56 new cases were diagnosed and 39 died. Despite the improvement in diagnosis and treatment, this population has a poor prognosis. About 59% have a 5-year survival after diagnosis and more than 5,000,000 of patients are alive in the United States [1,2,3].

The majority of patients with lung cancer experience burdensome symptoms associated to disease or treatments, which particularly occur in the advanced stage of their illness. Pain is one of the most distressing symptoms because of its debilitating effects, which produce depression and anxiety [4]. 

Patients with lung cancer develop complex and variable clinical conditions, depending on previous anticancer treatment received and the extension of disease. Pain if often a direct consequence of the malignancy and multifactorial in origin, so that patients may experience pain in different anatomical sites. Anticancer treatments may cause important pain syndromes, as it occurs after surgery or radiotherapy. Chest wall and vertebral bones are the most frequent sites of pain localization, although numerous syndromes have been identified [5].

About 90% of patients with lung cancer experience pain in the advanced stage of disease [5,6]. Pain adversely influences prognosis. The severity of cancer pain and high opioid dose are associated with shorter survival in advanced lung cancer, independently of other prognostic factors [7,8].

Cancer pain management is difficult and may sub-optimal in many circumstances. Different factors, including poor pain assessment, inadequate knowledge and sub-optimal prescription of analgesics and poor capabilities to manage opioid adverse-effects may result in an undertreatment [9].

Other than background pain, reported most hours of the day, cancer patients may experience peaks of pain intensity [10]. This type of pain, named breakthrough cancer pain (BTcP), is characterized by a peak in pain intensity, of short duration, that occurs spontaneously or is due to a specific trigger in patients who have a well-controlled pain for most hours of the day [11]. The temporal pattern of BTcP is capable of interfering with quality of life [12]. 

Indeed, data regarding the BTcP in patients with lung cancer is lacking. The primary outcome of this study was to assess BTcP characteristics in patients with lung in comparison with those of patients with other primary tumors. The secondary outcome was to assess the management of background pain and BTcP, patients’ satisfaction with medications used for BTcP and adverse effects associated with the use of drugs for BTcP.

## 2. Methods

This was a secondary analysis of a large, multicenter, national study of BTcP. Thirty-two centers were involved [13]. The local ethical committees approved the protocol and each patient provided a written informed consent. Patients were visited as outpatient, inpatient or day-hospital and were recruited in different settings, including oncology, palliative care, radiotherapy and pain clinic. Inclusion criteria were—age ≥ 18 years, a cancer diagnosis, the presence of stable and well-controlled background pain (pain intensity on a 0–10 numerical scale of ≤4), the presence of episodes of BTcP, with moderate-severe intensity and clearly distinguished from background pain, according to a pre-defined algorithm [11,14]. A condition of unstable or uncontrolled background pain (pain intensity on a 0–10 numerical scale of >4); episodes of low pain intensity (<5/10) and poor collaboration were exclusion criteria. Patients with lung cancer were selected from the original study. 

Age, gender and Karnofsky level were recorded. Average pain intensity (on a numerical scale 0–10) and analgesics prescribed for background pain and their doses (expressed as oral morphine equivalents (OME) [15] were recorded, as well the use of adjuvants. 

The characteristics of BTcP were collected—number and intensity (on a numerical scale 0–10), of BTcP episodes, predictability and triggering factors, BTcP onset (rapid or longer, ≤10 min or >10 min. respectively), duration of untreated BTcP episodes, the interference with daily life (nothing, a little bit, much, very much). BTcP medications, the mean time to meaningful pain relief after receiving the medication and satisfaction with BTcP pharmacological treatment (very satisfied, satisfied, not satisfied and neither satisfied nor dissatisfied), were assessed. Adverse effects specifically attributed to BTcP medications were recorded. 

### Statistics

Descriptive statistics for patients’ characteristics, basal pain and BTcP have been reported providing mean values and frequencies stratified by Lung cancer occurrence. Groups profiles, affected or not affected by lung cancer, have been compared, carrying out a multivariate Hotelling T2 test (α = 0.05). Post-hoc analysis performed pairwise comparisons Bonferroni’s adjusted for familywise error. Gender and age have been checked for confounding. A logistic regression provided different probabilities of Lung cancer occurrence conditioning on gender accordingly. Age was not statistically significant (*p* > 0.05). Lung cancer association patterns has been assessed using χ^2^ tests using personal, pain characteristics, BTcP, pain and BTcP medications. Independent *t*-tests for comparing groups have been performed where due, adjusting the degrees of freedom with the Satterthwaite correction to account for heteroscedasticity. Statistical software STATA (version 16, Statacorp, College Station, TX, USA) was used for the statistical analysis.

## 3. Results

Of the 4016 patients recruited in the original study, one-thousand-eighty-seven patients had lung cancer. The characteristics of patients with lung cancer and other tumors are described in Table 1. Pain mechanisms were mixed, nociceptive and neuropathic in 719 (66.1%), 276 (25.4%) and 92 (8.5%) patients, respectively. There was significant difference with other primary tumors (mixed 66.1% vs. 61.2%, nociceptive 25.4% vs. 30.7% and neuropathic 8.5% vs. 8.1%, *p* = 0.005). 

One thousand fifty-nine (97.4%) and 924 (85.0%) patients were prescribed opioids for background pain and for BTcP, respectively. The mean background pain intensity was 3.1 (Standard Deviation 1.78), which was higher than the pain intensity found in other tumors (*p* = 0.006). The mean OME was 65.9 mg/day. This dosage was lower than that reported in patients with other tumors (73.9 mg/day, SD 93, *p* = 0.005).

### 3.1. BTcP Characteristics

Patients with lung cancer reported a mean number of BTcP episodes of 2.4/day (SD 1.38, range 1–10). No differences in comparison with other primary tumors were found (*p* = 0.104).

The mean intensity of BTcP episodes was 7.6 (SD 1.27). A statistical difference with other tumors (7.4, *p* = 0.005) was found. The mean duration of an untreated BTcP episodes was 40.9 min (SD = 32.59). No statistical differences with other tumors were found (*p* = 0.174). 

BTcP was predictable in 343 patients (31.5%). No statistical difference with other tumors was found (*p* = 0.378). The main trigger of predictable BTcP was the movement (79.5%). Cough, ingestion of food, procedures and other causes were the other triggers (8.2%, 2.6%, 5.6% and 4.1%, respectively). When compared to other tumors, patients with lung cancer were more likely to have predictable BTcP with movement (79.5% vs. 57.7%) and cough (8.2% vs. 1.5%) and less likely to experience predictable BTcP related to ingestion of food (2.6% vs. 22.1%, *p* = 0.0001) and procedures (5.6% vs 6.0%) (*p* = 0.0001).

A short onset BTcP (≤10 min) was found in 771 patients (70.9%), while in 316 patients (29.1%) BTcP onset was longer (>10 min). No differences with other tumors were found (*p* = 0.099).

The mean time to meaningful pain relief after receiving a BTcP medication was 16.3 min (SD 14.52). No statistical differences with other tumors were found (*p* = 0.711)

BTcP interference with daily activity was mild, much and very much in 117 (10.9%), 653 (61.0%) and 297 (27.8%) patients, respectively. Statistical differences were found in comparison with other tumors, 14.7%, 56.2% and 28.8%, respectively (*p* = 0.009).

### 3.2. Analgesics Used for Background Pain

Other dugs administered for background pain were anti-inflammatory drugs (n.136, 12.5%), paracetamol (n.367, 33.8%); weak opioids (n.43, 4.0%); oral morphine (n.91, 8.4%), oral hydromorphone (n.33, 3.0%), oxycodone (n.207,19.0%); oxycodone/naloxone (n.342, 31.5%), tapentadol (n.67, 6.2%), parenteral morphine (n.46, 4.2%), methadone (n.8, 0.7%), transdermal fentanyl (n.268, 24.7%), transdermal buprenorphine (n.24, 2.2%). 

Eight-hundred-twelve patients (76.2%) were prescribed adjuvant drugs, including benzodiazepines (103, 9.5%), anticonvulsants (388, 35.7%), antidepressants (n.123, 11.3%), antiemetics (n.64, 5.9%), laxatives (n.182, 16.7%) and corticosteroids (n.490, 45.1%).

Patients with lung cancer more frequently used adjuvants (76.2% vs. 67.9%, *p* = 0.0001)—antiepileptics (35.7% vs. 28.7% *p* = 0.0001), antidepressants (11.3% vs. 8.7% *p* = 0.011) and corticosteroids (45.1% vs. 34.6% *p* = 0.0001) and less frequently antiemetics (5.9% vs. 10.2% *p* = 0.000) and benzodiazepines (7.1% vs. 10.3%, *p* = 0.013).

Patients with lung cancer were more frequently prescribed anti-inflammatory drugs (13.3% versus 6.4%, *p* = 0.0001 and *p* = 0.032 for females and males, respectively. No differences in the use of paracetamol were found (*p* = 0.242). 

Opioids used for background pain in patients with and without lung cancer are reported in Table 2. Oxycodone and tapentadol were more frequently used, while transdermal fentanyl was less frequently used in patients with lung cancer.

### 3.3. Analgesics Used for BTcP

Opioids prescribed for BTcP in patients with and without lung cancer are reported in Table 3. 

No differences in BTcP medication was found, unless for parenteral morphine which was less frequently used in patients with lung cancer (*p* = 0.002). No differences in the meaningful pain relief after BTcP medications were found (16.3 min, SD 14.5, versus 16.6 min, SD 13.88, *p* = 0.711).

### 3.4. Adverse Effects

Adverse effects due to BTcP medications were reported in 13 patients (2.5%). No differences with other tumors (2.7%) were found (*p* = 0.846). Thirteen patients reported adverse effects of mild intensity. No adverse effects of moderate or severe intensity were reported.

### 3.5. Satisfaction 

The majority of lung cancer patients were satisfied or much satisfied with BTcP medication (641 (61.5%) and 89 (8.5%), respectively). No differences with other primary tumors were found.

## 4. Discussion

The large sample of patients analyzed in this study allowed us to gather relevant information regarding the phenomenon of BTcP in patients with lung cancer. 

In comparison with patients with other tumors, patients with lung cancer had higher levels of background pain, while receiving lower opioid doses and more adjuvant drugs and anti-inflammatory drugs. BTcP intensity was higher in patients with lung cancer and resulted in a larger interference with daily activities. The main causes of predictable BTcP were movement and cough, suggesting some peculiarities of this population in comparison with population affected by other primary tumors. 

All these findings suggest that clinicians resort to other drugs to improve opioid analgesia, rather than increasing the doses of opioids, as pain would be less responsive to opioid drugs [9]. The use of adjuvant drugs is suggested by a recent study in which duloxetine and pregabalin were found to be effective in lung cancer patients with neuropathic pain [16]. Alternately, opioid therapy may not have been optimized These interpretations, however, deserves further studies with an appropriate design. The prevalence of predictable BTcP on movement or cough seems to reflect the typical local and metastatic expression of most lung cancers, also considering the prevalent somatic pain mechanism, suggesting a bone involvement with lung cancers.

Comparative data regarding BTcP in patients with lung cancer are limited. In a pioneer study performed in home care patients with lung cancer, somatic incident pain, which is a subtype of BTcP principally attributable to bone metastases, resulted to be a negative prognostic factor for pain control [6], as it occurred in the general cancer population [17,18,19]. In a retrospective study of 152 lung cancer patients, high pain intensity was associated with more BTcP episodes. Pain with high intensity, frequent BTcP episodes, the presence of bone metastases and neuropathic pain predicted a poor response to analgesics [8]. However, the retrospective nature study design and the lack of a predetermined definition of BTcP render the interpretation of this data difficult. In the present study, patients were selected only if their background pain was considered to be acceptable [13]. Nevertheless, patients with higher background pain intensity, even in the range of mild pain (0–4 on a numerical scale), were more likely to have more episodes with higher intensity, suggesting a possible optimization of background analgesia. 

There are some limitations of this study, as it was a secondary analysis of an original study performed in patients with a diagnosis of BTcP, according to a predetermined algorithm. Thus, the prevalence of BTcP in patients with lung cancer remains unknown. In a sample of advanced cancer outpatients with a prevalent diagnosis of lung cancer, the prevalence of BTcP was about 40% [20]. Moreover, the analgesic treatment and the choice of drugs was based on local policy. On the other hand, participating centers had a large experience in the management of cancer pain. Thus, data gathered from the present study reflects what occurs in the real world, providing a picture of the characteristics of such patients and the most common analgesic treatments employed for both background and BTcP The large sample provided reliable data. Lung cancer is a broad term that refers to different histologies and stages of cancer. Thus, additional information on subgroups of lung cancer may enhance the external generalizability of these findings. Specific studies assessing these aspects may add further information in patients with different types of lung cancer.

## 5. Conclusions

Patients with lung cancer have their own peculiarities, including higher basal and BTcP pain intensity and the more frequent use of adjuvant drugs for background pain. The most frequent triggers for predictable BTcP are movement and cough. The implication of the present findings is that patients with lung cancer have their own peculiarities in BTcP presentation. This knowledge may help clinicians provide the best management. Future studies should analyze the prevalence of BTcP in patients with different lung cancers as well as the optimal analgesic strategies for background pain and BTcP. 

## Figures and Tables

**Table 1:** Characteristics of patients with lung cancer and other primary tumors.

Characteristics		n (%) with Lung Cancer	n (%) with Other Tumors	*p*
Age yrs (mean SD)		66.0 (10.43)	64.1 (12.82)	0.0001
Gender (F/M)		338 (31.1%) 749 (68.9%)	1476 (50.4%) 1453 (49.6%)	0.0001
Karnofsky (mean SD)		62.1 (18.28)	61.7 (18.89)	0.1
Disease	Loco-regional	225 (20.7%)	511 (17.45%)	0.018
	Metastatic	862 (79.3%)	2418 (82.55%)	
Anticancer treatment	Disease-oriented	766 (72.7%)	2264 (80.0%)	0.0001
	Palliative Care	288 (27.3%)	566 (20.0%)	
Place of Visit	Outpatients	407 (37.4%)	971 (33.1%)	0.0091
	Day hospital	138 (12.7%)	324 (11.1%)	
	Home care	130 (12.0%)	447 (15.3%)	
	Hospice	26 (2.4%)	75 (2.6%)	
	Hospital inpatient	568 (35.5%)	112 (37.9%)	
Setting	Palliative care	169 (15.5%)	551 (18.8%)	0.028
	Oncology	568 (52.3%)	1519 (51.9%)	
	Pain therapy	346 (31.8%)	838 (28.6%)	
	Radiotherapy	4 (0.4%)	21 (0.7%)	
Mean PI		3.1 (SD 1.78)	2.9 (SD 1.84)	0.006
Mean OME		65.4 mg/day (SD 78.92),	73.9 mg/day (SD 93.68)	0.005

PI = background pain intensity. OME = oral morphine equivalents.

**Table 2:** Opioids used for background pain in patients with and without lung cancer.

Opioids	n (%) with Lung Cancer	n (%) with Other Tumors	*p*
Oral morphine	91 (8.4%)	238 (8.1%)	0.801
Oral hydromorphone	33 (3.0%)	95 (3.2%)	0.739
Oxycodone	207 (19.0%)	457 (15.6%)	0.009
Oxycodone/naloxone	342 (31.5%)	810 (27.6%)	0.018
Tapentadol	67 (6.2%)	128 (4.4%)	0.019
Parenteral morphine	46 (4.2%)	147 (5.0%)	0.169
Methadone	8 (0.7%)	35 (1.2%)	0.209
Transdermal fentanyl	268 (24.7%)	834 (28.5%)	0.016
Transdermal buprenorphine	24 (2.2%)	97 (3.3%)	0.069

**Table 3:** Opioids used for breakthrough cancer pain (BTcP) in patients with and without lung neck cancer.

Opioids	n (%) with Lung Cancer	n (%) with Other Tumors	*p*
OTFC	33 (3.0%)	97 (3.3%)	0.661
FBT	112 (10.3%)	323 (11.0%)	0.512
FBST	186 (17.1%)	384 (13.1%)	0.001
FPNS	228 (21.0%)	579 (19.8%)	0.396
INFS	13 (1.2%)	27 (0.9%)	0.437
Oral morphine	140 (12.9%)	423 (14.4%)	0.205
Parenteral morphine	59 (5.4%)	234 (8.0%)	0.006
